# Constitutive expression of a grapevine polygalacturonase-inhibiting protein affects gene expression and cell wall properties in uninfected tobacco

**DOI:** 10.1186/1756-0500-4-493

**Published:** 2011-11-13

**Authors:** Erik Alexandersson, John VW Becker, Dan Jacobson, Eric Nguema-Ona, Cobus Steyn, Katherine J Denby, Melané A Vivier

**Affiliations:** 1 Institute for Wine Biotechnology, Department of Viticulture and Oenology, Faculty of AgriSciences, Stellenbosch University, Stellenbosch, South Africa; 2 Department of Molecular and Cell Biology, University of Cape Town, Private Bag, Rondebosch, Cape Town, South Africa; 3 School of Life Sciences and Warwick Systems Biology Centre, University of Warwick, Wellesbourne Campus CV35 9EF, UK; 4 Department of Plant Protection Biology, Swedish Agricultural University, P.O. Box 102, SE-230 53 Alnarp, Sweden; 5 African Centre for Gene Technologies, Experimental Farm, University of Pretoria, Lynnwood Ridge, Pretoria, South Africa

## Abstract

**Background:**

Polygalacturonase-inhibiting proteins (PGIPs) directly limit the effective ingress of fungal pathogens by inhibiting cell wall-degrading endopolygalacturonases (ePGs). Transgenic tobacco plants over-expressing grapevine (*Vitis vinifera*) *Vvpgip1 *have previously been shown to be resistant to *Botrytis *infection. In this study we characterized two of these PGIP over-expressing lines with known resistance phenotypes by gene expression and hormone profiling in the absence of pathogen infection.

**Results:**

Global gene expression was performed by a cross-species microarray approach using a potato cDNA microarray. The degree of potential cross-hybridization between probes was modeled by a novel computational workflow designed in-house. Probe annotations were updated by predicting probe-to-transcript hybridizations and combining information derived from other plant species. Comparing uninfected *Vvpgip1*-overexpressing lines to wild-type (WT), 318 probes showed significant change in expression. Functional groups of genes involved in metabolism and associated to the cell wall were identified and consequent cell wall analysis revealed increased lignin-levels in the transgenic lines, but no major differences in cell wall-derived polysaccharides. GO enrichment analysis also identified genes responsive to auxin, which was supported by elevated indole-acetic acid (IAA) levels in the transgenic lines. Finally, a down-regulation of xyloglucan endotransglycosylase/hydrolases (XTHs), which are important in cell wall remodeling, was linked to a decrease in total XTH activity.

**Conclusions:**

This evaluation of PGIP over-expressing plants performed under pathogen-free conditions to exclude the classical PGIP-ePG inhibition interaction indicates additional roles for PGIPs beyond the inhibition of ePGs.

## Introduction

Polygalacturonase-inhibiting proteins (PGIPs) are extracellular leucine-rich repeat (eLRR) proteins present in plants with recognition and inhibition capabilities towards fungal endopolygalacturonases (ePGs; [1]). ePGs are capable of hydrolyzing the homogalacturonan component of plant cell wall pectin and are among the first enzymes to be secreted during fungal infection. These enzymes play a major role in the virulence of several phytopathogenic fungal and bacterial species [2-5]. Most notably, two ePGs of the necrotrophic fungus *Botrytis cinerea *are required for its full virulence on different plant hosts [4,5].

PGIPs differentially inhibit ePGs from not only a diverse range of fungi, but also different ePG isoforms from the same fungus [1]. This is also well illustrated for grapevine VvPGIP1 and ePGs from *Aspergillus niger *and *B. cinerea*, where differential inhibition towards these ePGs was observed in *in vitro *assays [6]. Further *in vitro *evidence suggests that the interaction and resultant inhibition of ePG by PGIP leads to prolonged existence of molecules with the ability to up-regulate the plant's defense response [7]. Thus, it has been suggested that PGIPs protect plants from fungal infection not only by inhibiting fungal macerating enzymes and thereby directly limiting tissue damage, but also by switching on plant defense signaling pathways [8].

Although these *in vitro *experiments have contributed significantly to our understanding of the PGIP-ePGs interaction, recent findings have highlighted the need to better understand the *in planta *roles of PGIP. For example, it was shown that over-expression of *Vvpgip1 *reduces the symptoms of BcPG2 from *B. cinerea *in tobacco leaves, without any evidence for an *in vitro *interaction [9]. From this work it appeared that the *in vivo *environment provided a context for this specific PGIP-ePG pair that could not be created *in vitro*. It was proposed that VvPGIP1 might bind to pectin as was shown for bean PGIPs [10] and that VvPGIP1 did not directly inhibit BcPG2, but perhaps rather shielded the most exposed and vulnerable positions in the pectin, thereby indirectly protecting against ePG actions [9].

Numerous studies have reported that high levels of *pgip *gene expression reduce the susceptibility towards *B. cinerea*, confirming the importance of PGIP in plant defense. These include over-expression of the pear *pgip *gene in tomato [11] and grapevine [12], *Arabidopsis pgip *genes over-expressed in *Arabidopsis *[13], a bean *pgip *gene in tobacco [14] and the grapevine *pgip *gene in tobacco [6]. In contrast, silencing of *Arabidopsis Atpgip1 *led to enhanced susceptibility [15]. Recently, Veronico et al. [16] reported that the expression level and pattern of a native pea *Pvpgip *could be linked to the degree of resistance to cyst nematode infections. Interestingly, no ePG transcripts could be derived from the cyst nematode nor was a correlation seen between PGIP expression and a native pea ePGs, suggesting that PGIPs have a role in plant-pathogen interactions outside of the classical PGIP-ePG inhibition. More light was shed on the *in planta *role of PGIPs when Kanai et al. [17] presented data from *Arabidopsis *knock-out mutants and over-expressing lines, indicating that *Atpgip1 *transcripts prolonged seed germination by influencing pectin degradation in the seed coat. Furthermore, the authors presented evidence that *Atpgips *are under the control of ABI5, a bZIP-type transcription factor that binds to ABRE elements.

Tobacco is a commonly used species to monitor plant-pathogen interactions and is suitable for PGIP over-expression studies since it has negligible PGIP activity against *Botrytis *ePGs [6,18]. We have previously demonstrated that transgenic tobacco plants over-expressing *Vvpgip1 *leading to an increased PGIP enzyme activity are less susceptible to *B. cinerea *infection in both detached leaf and whole-plant time-course fungal infection assays [6]. These lines are considered to be PGIP-specific resistant lines (i.e. the resistance phenotype could be correlated with VvPGIP1 over-expression, activity levels as well as ePG-inhibition profiles) and as such provide a valuable genetic resource to study the possible role(s) of PGIPs in plant defence.

To this end, global gene expression, hormone profiling and subsequently, cell wall analysis were conducted on two *Vvpgip1 *over-expressing lines with previously characterized resistance phenotypes in the absence of an infecting pathogen. The presence of PGIP, under these pathogen-free conditions, caused altered expression of genes related to a range of mechanisms, including primary metabolism, cell wall organization and metabolism, water transport, photosynthesis and defense responses. An *in silico *cross-species co-expression analysis revealed that many of these gene families were also co-expressed in *Arabidopsis*. Analysis of cell wall components of the plants over-expressing *Vvpgip1 *showed a composition similar to wild-type (WT) with only a slight decrease in rhamnose content. However, the change in gene expression was accompanied by higher lignin content, increased level of auxin and, following *Botrytis *infection, a stronger jasmonic acid response. Furthermore, transcriptional down-regulation of a group of xyloglucan endotransglycosylase/hydrolases (XTHs) with key roles in cell wall restructuring and remodeling led to a reduced XTH enzyme activity in the uninfected transgenic tobacco. Taken together, these findings suggest that altered PGIP expression has an effect on the cell wall structure, also affecting fundamental mechanisms such as primary metabolism.

## Materials and methods

### Plant material and growth conditions

Transgenic *Nicotiana tabacum *SR1 (Petit Havana) *Vvpgip1 *line 37 and 45 described in [6] and WT plants were grown in a mixture of soil and peat moss (Jiffy Products International AS, Norway) at 24°C and 55% relative humidity with a light intensity of 120 μmol m^2 ^s^-1 ^over a 16 h light period. Plants were supplemented with liquid organic fertilizer every two weeks (Nitrosol^®^, Fleuron (Pty) Ltd, South Africa). Leaf material from leaf positions three to five, where leaf three is the youngest and first fully expanded leaf, from healthy 6 to 8-week-old transgenic and control tobacco plants was flash frozen in liquid nitrogen and stored at -80°C for RNA extraction, lignin analyses and phytohormone profiling. For hormone profiling during *Botrytis *infection, *B. cinerea *pathogenic cultures were prepared as described in [6]. Plants were grown in 100% relative humidity and infections performed with four inoculation spots per leaf of 5 μL of a *B. cinerea *spore suspension (1 × 10^3 ^spores in a 50% grape juice medium per spot). Infections were allowed to progress for 0, 18, 24 and 30 h before tissue immediately surrounding and including the infection spots (15 mm diameter) were harvested (separate plants per infection time point were infected and harvested to eliminate wound-response effects). For cell wall component analysis and XTH activity assays, transgenic lines were first established on MS medium supplemented with kanamycin [19] and then transferred to soil and grown under natural light conditions at a controlled temperature. Leaves from leaf position 3, 4 and 5 were harvested when the plants reached the six leaf stage.

### RNA extraction and microarray analyses

For microarray analysis leaves of the same age and position from individuals of two transgenic lines were compared to the same WT plant. RNA from 0.5 to 1 g finely ground plant material was extracted using a sodium perchlorate extraction buffer (5 M sodium perchlorate, 0.3 M Tris-HCl pH 8.3, 8.5% polyvinylpolypyrollidone (PVPP), 2.0% PEG 4000, 1.0% β-mercapto-ethanol, 1.0% SDS). After shaking for 30 min at room temperature samples were centrifugated and plant debris was removed by passing the supernatant through a syringe plugged with cotton wool. Several phenol/chloroform extractions were performed before precipitating the RNA with 2.5 M LiCl at -20°C overnight. The pellet was washed with 70% ethanol and the resuspended RNA was purified using the RNeasy Mini Kit (Qiagen GmbH, Hilden, Germany). RNA integrity was ensured on 1.2% formaldehyde gels and purity was determined by 260/230 and 260/280 absorbance ratios (>2).

Twenty-five μg RNA was used in each cDNA synthesis reaction in a total volume of 30 μL. Before denaturation at 70°C for 10 min, 2 μL of oligo d(T) primers (500 μg/mL) were added. After denaturation, first strand buffer and DTT were added according to the manufacturer's recommendation (Invitrogen, Carlsbad, CA, USA). A 2:3 (aa-dUTP:dTTP) aminoallyl-dNTP (Ambion, Austin, TX, USA) mix was added (0.5 mM each of dATP, dCTP and dGTP; 0.2 mM aa-dUTP and 0.3 mM dTTP) before incubation at 46°C for 2 min, after which 200 U of SuperScript III Reverse Transcriptase (Invitrogen, Carlsbad, CA, USA) was added. The same amount of enzyme was added following incubation for 4 h at 46°C, after which cDNA synthesis proceeded overnight.

RNA was hydrolyzed by 10 μL 1 M NaOH and 0.5 M EDTA and incubated at 65°C for 15 min. To neutralize the pH, 10 μL 1 M HCl solution was added before unincorporated aminoallyl dUTP and free amines were removed using the RNeasy Mini Kit. cDNA was quantified using a Nanodrop and dried down to volumes of less than 1 μL in a vacuum dryer. Five μL of 0.1 M Na_2_CO_3_, pH 9.0, was added to the cDNA, and the mixture incubated at 37°C for 10 min. Cy3 or Cy5 (Amersham Biosciences, Buckinghamshire, UK) ester was added and the coupling allowed to proceed for 1 h in the dark at room temperature. Uncoupled dyes were removed by purification with the RNeasy Mini Kit. Probe labeling efficiency of Cy-esters was estimated by measuring the absorbance at 550 nm and 650 nm. Similar amounts of labeled samples were hybridized on TIGR 10 K potato microarrays (version 3). Pre-hybridization was done under lifterslips (Erie Scientific, Portsmouth, NH, USA) with a pre-warmed solution containing 5X saline-sodium citrate (SSC) buffer, 0.1% SDS and 1% BSA at 42°C for at least 30 min. The slides were then washed in five times of deionized water and finally briefly submerged in ethanol before short centrifugation. The combined Cy-labeled probes (28 μL) were mixed with 30 μL of a 2X hybridization buffer (50% formamide, 5X SSC and 0.2% SDS), 1 μL each of COT1 DNA (1 μg/uL) and poly(A)-DNA (12 μg/μL) added, for a total volume of 60 μL, denatured at 90°C for 3 min, and subsequently applied to the slide using lifterslips. Slides were incubated for 16 h at 42°C in hybridization chambers (ArrayIt, Telechem International, Sunnyvale, CA, USA) and successively washed in low stringency (2X SSC, 0.5% SDS; heated to 55°C), medium stringency 0.5X SSC) and high stringency (0.05X SSC) wash buffers for 5 min each and then briefly submerged in ethanol prior to short centrifugation. Slides were scanned with an Axon GenePix 4000A scanner using the GenePix 5.1 software (Molecular Devices, Sunnyvale, CA, USA).

Three microarray slides in total were hybridized: two slides using *Vvpgip1 *line 37 and WT, with a dye swap included to account for dye bias; and one slide with *Vvpgip1 *line 45 and WT. The result files were analyzed using the Limma package in R 2.9.1 [20]. Spots flagged as "bad" by the GenePix software were removed from further analysis. Background correction was done by the normexp method [21] with an offset of 50 to avoid negative intensities and normalization was then done by print-tip loess. The duplicateCorrelation function was used to estimate a common value for within-array duplicated spots [21]. Finally, fold changes and standard errors were obtained by fitting a linear model to each gene and standard errors were smoothed by empirical Bayes. Genes with a p-value below 0.05 after false discovery rate (FDR) control were regarded as significant. The microarray data was deposited in GEO (GSE26324).

### Real-time quantitative PCR

For RT-qPCR analyses RNA was DNase treated (Qiagen GmbH, Hilden, Germany). cDNA was synthesized using SuperScript III Reverse Transcriptase according to the manufacturer's specifications (Invitrogen, Carlsbad, CA, USA) using 1 μg of RNA. Both oligo d(T) and random primers were added to obtain full length cDNA. cDNA samples were diluted 1:25 in dH_2_O before 5 μL of sample was added to 15 μL of LightCycler FastStart DNA Master SYBR Green I mix, prepared according to the manufacturer's recommendations (Roche Diagnostics GmbH, Mannheim, Germany) with a final primer concentration of 500 nM. RT-qPCR was performed using the LightCycler Instrument (Roche Applied Science).

Transcript specific primers were designed using Primer Express (Applied BioSystems) with default settings. Primer sequences for the tobacco genes xyloglucan endotransglycosylase (*XTH*, Genbank Acc AB017025.1) and tubulin used as reference gene (*TUB*, Genbank Acc AB052822) were: *XTH *forward 5'-AGTCCAAGTTTGTAACACC-3' and reverse 5'-TCTGTCCTTAGTGCATTCTG-3', amplification product 175 bp; *TUB *forward 5'-TCTGGCTGCTCTGGAAA-3' and reverse 5'-GCATACAAGACACCATCAAAT-3', amplification product 197 bp. cDNA amplification conditions were as follows: denaturation at 95°C for 10 min, followed by 45 cycles of denaturation, 95°C for 10 s; primer annealing at 58°C for 10 s and primer extension at 72°C for 8 s, during which data acquisition was performed. Melting curve analysis was performed by increasing the temperature by 0.1°C/s in the interval 65°C to 95°C. PCR efficiencies for each sample were calculated using LinRegPCR software [22]. The efficiencies were used to calculate relative expression in a mathematical model [23].

### XTH activity determination

To determine the XTH activity in tobacco leaves, a dot-blot assay based on the method described in [24] was used. Whatman 3MM paper was coated with 1% (w/v) Tamarind seed xyloglucan (Megazyme) dissolved in aqueous 0.5% (w/v) 1,1,1-trichloro-2-methylpropan-2-ol (Sigma-Aldrich, Steinheim, Germany). Eight leaves were harvested per leaf position and frozen in liquid nitrogen. Two leaves from two individual plants were pooled to ensure adequate amounts of material. This was done for all the lines tested and for leaf position three, four and five. Four extractions were done per biological repeat and each extract's activity was measured in triplicate as described above. Boiled extracts served as negative control, while a batch of cauliflower extract served as positive control for each assay [24].

Total protein was extracted from 20 mg of freeze-dried material for 6 h in 1 mL 50 mM NaOAc, pH 5.5, with gentle agitation at 8°C. The supernatant was collected after centrifugation at 9600*g *for 20 min at 4°C. The protein concentration was determined by the Bradford method [25] with bovine serum albumin as standard. 10 μl of each extract containing 6 nmol of the SR-conjugate (XLLGol-SR) was spotted on the 3MM paper at 4°C and then incubated at room temperature for 12 h between cellulose acetate sheets and several tissue papers to apply an even load. It was then washed for 4 h in 100 mL of 50% ethanol, rinsed in acetone and dried.

The fluorescence was measured with the IVIS^® ^100 Imaging System (Caliper Life Sciences) using the DsRed filter (570 nm excitation, 615 nm emission) with a 1 s exposure. The Living Image software (Caliper Life Sciences, Hopkinton, MA, USA) was used to identify regions of interest (ROI) and quantify the total efficiency per cm^2 ^for each fluorescent spot. Background fluorescence was subtracted from all values, which were then normalized to total protein.

### Cell wall analysis

Cell wall materials were extracted according to a protocol modified from [26]. Briefly, frozen tobacco leaves were immersed in liquid nitrogen and ground with a mortar and a pestle into a fine powder. Ground material was subsequently boiled with 80% ethanol for 20 min, washed in methanol:chloroform (1:1) for 24 h and finally washed with methanol. Tobacco tissues were left to incubate in methanol:chloroform (1:1) for another 24 h, due to the high level of lipids and others fatty materials (e.g. waxes) present. The remaining material (also called alcohol-insoluble residues, AIR) was dried in an oven at 70°C.

Total cell wall monosaccharide composition was determined on transgenic tobacco over-expressing *Vvpgip1 *line 37 and WT. AIR (2 to 4 mg) from different leaf position (3 to 5) were hydrolyzed using 2 M trifluoroacetic acid (2 M TFA, 2 h at 110°C), followed by a 18 h methanolysis at 80°C with dry 2 M methanolic HCl. The generated methyl glycosides were converted into their TMS derivatives at 80°C and separated by gas chromatography (Hewlett Packard 5890 series II). The gas chromatographer was equipped with a flame ionization detector. The oven temperature program was 2 min at 120°C, 10°C/min to 160°C, and 1.5°C/min to 220°C and then 20°C/min to 280°C. Monosaccharides were identified based on their retention time and quantified by determination of their peak areas. The GC-FID was calibrated by using a range of increasing concentration of a mixture of our nine standard sugars and the sugar composition was expressed in molar percentage of monosaccharide. Myo-inositol (90 μL of 1 mg/mL solution) was used as an internal standard. In the final analysis, glucose was removed because of likely contamination of the cell wall fractions by starch-derived glucose.

### Histochemical lignin assays and lignin quantification

The lignin content of the WT and *Vvpgip1 *lines 37 and 45 was estimated in leaf sections with potassium permanganate staining followed by transmission electron microscopy according to [27]. Additionally, the lignin content in the stems of the same lines was estimated by staining with a solution of phloroglucinol according to [28].

Quantification of lignins in the WT and transgenic lines *Vvpgip1 *line 37 and 45 was done using the acetyl bromide method as described in [29], with some modifications as described below. Dried tobacco leaf material (leaves three to five of eight week old plants) was utilized to isolate AIR, consisting of cell wall material. Leaf tissue was ground in liquid nitrogen and extracted twice with 80% aqueous methanol following homogenization. Following centrifugation at 12 000x*g *for 10 min, the pellets were washed three times with 96% ethanol and twice with a solution of 96% ethanol:hexane (2:1). The resulting AIR pellets were dried overnight at 70°C. Five to ten mg of the AIR was used to determine the percentage of lignin contained therein. The AIR was washed with 25% acetyl bromide (in acetic acid), after which it was incubated in 1 mL of the same solution at 70°C for 30 min. The mixture was cooled to room temperature, and 0.9 mL of NaOH and 0.1 mL of hydroxylamine hydrochloride (0.1 M) added. The volume was subsequently adjusted to 10 mL with acetic acid. The solution was incubated overnight and the absorbance measured at 280 nm with a procedural blank. The lignin content of the samples was calculated as follows: % lignin content = (absorbance × 100)/(SAC × AIR (g l^-1^)); where SAC is the specific absorption coefficient of lignin, for which the value of 20 gl^-1 ^cm^-1 ^was used.

### Analysis of phytohormones using GC/MS

Leaf tissue flash frozen in liquid nitrogen was extracted according to the method of Schmelz et al. [30], with some modifications as described below. Approximately 100 mg of tissue was ground to a fine powder prior to the addition of extraction solvent (n-propanol/water/HCl) and internal standard (*o*-anisic acid), of which 30 ng per sample was added. Samples were vortexed to ensure homogenization prior to partitioning with dichloromethane. For the conversion of phytohormone acids to their corresponding methyl esters, the organic phase (dichloromethane/propanol) was derivatized in 4 mL glass vials for 30 min using 4 μL of a 2 M trimethylsilyldiazomethane solution in hexane. The activity of the derivatization agent was subsequently quenched with 4 μL of a 2 M acetic acid solution, also in hexane.

Vapor phase extraction of the derivatized organic phase proceeded according to [30], with the exception that commercially available Super Q filters were used (Analytical Research Systems, Inc., Gainesville, FL, USA). Briefly, the derivatized sample was evaporated at 70°C and passed through a Super Q filter under a N_2 _flow of 500 mL/min. To ensure complete vaporization of less volatile compounds the vial was subsequently heated to 200°C for 2 min while passing the vapor through the filter.

The analytes were eluted from the Super Q filter with 150 μL CH_2_Cl_2 _and analyzed by a Trace Gas Chromatograph (GC) (ThermoFinningan, Milan, Italy) coupled to a Trace Mass Spectrometer (MS) (Thermo MassLab, Manchester, UK). GC/MS conditions were amended from that described in [30]. Briefly, 2 μL of the dichloromethane (BDH, Poole, England) eluent was injected in the split/splitless injector of the GC, operated in the splitless mode (purge time 3.5 min, 50 mL/min) at 280°C. Compounds were separated on a Factor Four VF5-MS capillary column (Varian, Palo Alto, CA, USA) with dimensions 30 m 1. × 0.25 mm i.d. × 0,25 μm f.t. Flow of the carrier gas (Helium) through the column was 0.7 mL/min in the constant flow mode. The oven program used was 40°C, hold 1 min, ramp 15°C/min to 250°C, hold 5 min, ramp 20°C/min, and hold 2 min. In order to avoid carryover a post-run was performed after each analysis at 280°C under a head-pressure of 300 kPa. The temperature of the MS interface was kept at 280°C and the source at 200°C. The MS-detector was operated in Electron Impact (EI) mode at 70 eV and Selected Ion Monitoring (SIM) mode. The electron multiplier voltage was set at 500 V. Three carboxylic acid methyl ester analytes were detected and quantified using SIM with retention times and ion mass to charge ratios (m/z) as follows: methyl salicylate (8.39 min, m/z 92, 120, 152); methyl jasmonate (12.38 min, m/z 83) and methyl indole-3-acetate (13.81 min, m/z 130). The internal standard, *o*-anisic acid methyl ester was eluted at 9.70 min with m/z 92, 120 and 152.

For quantification the internal standard method was used. Calibration curves were constructed for each analyte over the range from 2 to 200 ng.mL^-1^. The regression equations and their correlation coefficients obtained for SA, MeJA and IAA are detailed respectively: y = 0.0370x+0.2511 (r^2 ^= 0.9924), y = 0.0087x+0.0303 (r^2 ^= 0.9954) and y = 0.0120x + 0.0238 (r^2 ^= 0.9964). The limit of quantification (LOQ) was established to be 2 pg for all analytes. Salicylic acid, indole-3-acetic acid, methyl jasmonate, *o*-anisic acid, trimethylsilyldiazomethane, hexane and 1-propanol were purchased from Sigma-Aldrich (Steinheim, Germany).

### Bioinformatics workflow for probe evaluation and annotation

The interpretation of the microarray results was challenged by the potential ambiguity in probe to transcript hybridizations and incomplete annotations of the genes of the two plant species involved.

#### Probe Specificity and annotation

BLAST was used to map all of the extant *N. tabacum *ESTs (PlantGDB-derived unique transcripts version number 173a; [31]) to the probes they would likely hybridize to on the TIGR 10 K potato microarray and the result stored as a graph denoting the relationships between potential transcripts and probes. A threshold of 80% identity over at least a 100 bp region was used to identify transcripts that would be likely to hybridize to microarray probes.

As annotated genome sequences are available for neither *S. tuberosum *nor *N. tabacum*, EST datasets were used for annotation based analysis. However, the annotation was incomplete and out of date for both the EST datasets for *S. tuberosum *(from which the probes were designed) and *N. tabacum*. Prot4EST [32], which encompasses BLASTX [33], ESTScan [34] and DECODER [35] was modified in order to run on 100 × 2.83Ghz cores high performance computing architecture (Stellenbosch University) and the EST sequences from both *S. tuberosum *and *N. tabacum *translated into their corresponding protein sequences. The PLAZA [36] protein translations and associated GO and Interpro annotation for the complete genomes of *Ostreococcus lucimarinus, Chlamydomonas reinhardtii, Physcomitrella patens, Sorghum bicolor, Oryza sativa, Vitis vinifera, Populus trichocarpa Carica papaya *and *Arabidopsis thaliana *were downloaded. BLASTP and OrthoMCL [37] were used to create orthologous clusters based on sequence similarity of all proteins resident in PLAZA with the translated *S. tuberosum *and *N. tabacum *ESTs. GO and Interpro annotations found for members of the resultant orthologous clusters were projected onto the orthologous members for *S. tuberosum *and *N. tabacum *proteins. Subsequently, with the use of the probe-to-transcript graph described above, annotations from both *S. tuberosum *and *N. tabacum *were assigned to the corresponding probes.

A graph structure was created to represent all of the resulting clusters, their associated annotation, assignment to probes and corresponding expression values. The resulting graph structure stored in XGMML and Cytoscape [38] were used for visualization and querying. In order to create Additional file 1, a Perl program was written to parse the graph structure, extract all connected components (e.g. clusters described above), and parse the BLASTP results to extract the most similar protein in *Arabidopsis *or rice (if no *Arabidopsis *match was found).

#### GO Enrichment

Significantly differentially expressed probes were determined by Limma as described above. The projected annotation for the differentially expressed probes was then analyzed for Enrichment of Gene Ontology Terms by GOEast using default settings [39].

#### Pathway and cross species co-expression analysis

BLAST was subsequently used to map all of the sequences associated with *Arabidopsis *gene identifiers onto specific probesets on the ATH1 Affymetrix microarray (thus yielding a direct map of *S. tuberosum *orthologous clusters to *Arabidopsis *probesets). A program was written in Perl to download the expression data from over 1700 *Arabidopsis *Affymetrix microarrays available at CressExpress [40]. Vectors of each probeset in the Affymetrix microarrays were created, including expression information from each microarray in the collection. Each of the differentially expressed *S. tuberosum *orthologous clusters were then analyzed for co-expression in *Arabidopsis *using these vectors and a Hierarchical Clustering Algorithm testing Pearson coefficient correlations thresholds of 0.05, 0.1 or 0.2 to select the clusters representing the highest levels of co-expression.

Visualization of affected genes divided into metabolic pathways or other processes was done by MapMan using a mapping file previously developed for the potato TIGR 10 K microarray [41].

### Statistical analysis

Excel (Microsoft) and GenStat (VSN International) were used to generate Student's t-tests and ANOVAs as indicated in the text.

## Results and discussion

Transgenic plants from various species with increased levels of polygalacturonase-inhibiting proteins (PGIPs) are known to have better protection against pathogenesis by *B. cinerea *and Pierce's disease [11-14], whereas *Arabidopsis *plants with *pgip *silencing show increased susceptibility towards *Botrytis *infection [15]. Clearly, PGIP expression levels affect pathogen infection and host-pathogen interaction.

The two *Vvpgip1*-overexpressing tobacco lines *Vvpgip1 *line 37 and 45, which are the focus of this study, were selected for further profiling from a population of transgenic lines considered to have PGIP-specific resistance phenotypes, since *pgip *over-expression, PGIP activity and ePG inhibition correlated with resistance against *B. cinerea *as shown Joubert et al. [6]. In order to shed more light on the possible processes associated to PGIP expression *in planta *and the role of PGIP leading to increased pathogen resistance, gene expression and hormone profiling was conducted. These analyses together with biochemical analyses of the cell wall confirmed that healthy un-infected PGIP transgenic plants have altered gene expression, hormone levels and cell wall structure.

### Global gene expression profiling

When the study was initiated, no microarray platform for tobacco was available. Instead, genome-wide expression in tobacco plants was monitored by the TIGR 10 K potato microarray, since these two species in the *Solanaceae *family have a high degree of sequence conservation and the heterologous hybridization system using microarray probes for potato ESTs has previously been used and evaluated [42]. Unfortunately, the latest annotation for the potato microarray was created in 2006, and thus did not contain the latest information on gene function. Further complicating matters is the potential ambiguity introduced by cross-hybridization that is bound to occur under heterologous hybridization.

**Figure 1:**
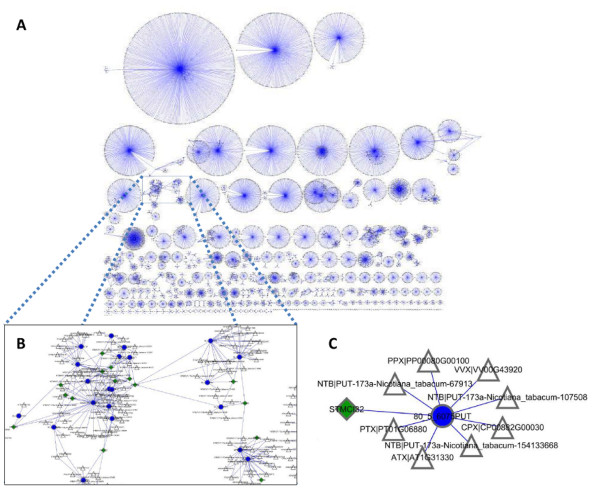
**Example of the graph structure**. The graph structure was used to assess possible cross-hybridization and probe redundancy as well as to improve annotation of probes. **a**. Graph structure of probes with significant change in signal in *Vvpgip1 *line 37. **b**. Detailed view of part of the graph structure showing differentially expressed probes in *Vvpgip1 *line 37. **c**. Example of an orthologous cluster. Probes are marked as *green squares, blue circles *represent a unique identifier for the orthologous cluster and matching transcripts are included as unfilled triangles.

To address these issues, the degree of cross-hybridization of tobacco transcripts to the potato probes was modeled by sequence similarity analysis of all of the tobacco ESTs as compared to the sequences reported for each probe on the microarray. This was achieved by generating a graph structure based on probe sequences and tobacco ESTs, which was used to estimate probe specificity and redundancy. Figure 1 gives an example overview of the graph structure, which were also used to update and re-annotate the microarray's probe annotations based on the tobacco transcripts likely to hybridize to a certain probe and by creating orthologous clusters based on the comparisons to nine sequenced plant genomes included in PLAZA [36]. By this approach a table of GO terms associated to each probe was generated and subsequently used for GO term enrichment analysis (Additional file 2). This file can be downloaded for future use when analyzing gene expression data generated by the TIGR 10 K potato (version 3) microarray. Heterologous hybridization on arrays has been estimated before [43,44]. However, we are unaware of a study that also takes into account the affect of cross-hybridization on the probe annotation of the microarray and subsequent GO term enrichment analysis.

By processing expression data in Limma a total of 318 probes were found to be differentially expressed with a false discovery rate (FDR) of ≤ 0.05 in both *Vvpgip1 *line 37 and 45 in comparison to WT (Table 1). Because the two transgenic lines most likely have different genotypic backgrounds due to the independent insertion events during transformation, we regard this approach to be technically more correct than considering the two lines as biological replicates by combining the expression data prior to Limma analysis. It is also a more conservative approach since considering the lines as biological replicates would almost double the number of probes (735) showing significant differential expression. A complete list of probes with significant difference in expression is included as Additional file 1 and a selection in Additional File 3. The majority of the probes were down-regulated, whereas only 58 were up-regulated in comparison to WT plants (Table 1). The remaining 41 probes showed opposite regulation in the two lines. The oppositely regulated probes can be a result of positional effects related to the insertion of the *Vvpgip1* expression cassette in the tobacco genome and thus reflect true differences between the transgenic lines. Alternatively, it might arise from rapid fluctuation in the abundance of certain transcripts caused by PGIP over-expression.

**Table 1:** Differentially expressed probes in *Vvpgip1 *line 37 and 45 in comparison to WT (FDR, *p < 0.05*)

*Vvpgip1 *line 37	*Vvpgip1 *line 45	Probes
Down	Down	219

Down	Up	16

Up	Down	25

Up	Up	58

	**Total**	**318**

Differentially expressed probes were analyzed by examining the graph structure of orthologous clusters and by examining top hits based on sequence identity (Figure 1; Additional file 1). Because of the probe ambiguity and the likelihood that many probes bind different transcripts, the exact number of differentially expressed genes monitored could not be determined. In fact, since the tobacco genome still remains un-sequenced, probe redundancy and specificity can only be estimated at this point. However, based on the graph structure we approximate that the 318 differentially expressed probes correspond to ca 250 genes.

A manual categorization of clusters and probes were done with the help of the MIPS 2.0 database [45], and probes were consequently divided into functional groups according to Additional file 1. Probes for which no sequence information was available are also listed if a significant change in signal intensity was observed. More than 15% of all clusters identified could not be functionally classified since the level of identity to other sequences was too low or no informative annotation could be inferred from related genes.

Even if the changes in expression were generally subtle there was an over-representation of certain functional groups among the differentially expressed genes when dividing genes by GO terms or MapMan categories. GO term enrichments were based on probes with a significant change in signal in both transgenic lines in comparison to the wild-type (WT). The full graphical representation of enriched terms can be found in Additional file 4.

Four broad groups of affected probes fall under the categories of cell wall biogenesis and organization, carbon metabolism, photosynthesis and stress defense signaling, whereas more specific groups of interest would be glucan and polysaccharide metabolic processes, water transport as well as response to auxin and brassinosteroid stimuli and to cyclopentones. Biosynthesis of jasmonic acid (JA) requires several cyclopentenone precursors and these have been suggested to be able to fulfill some of the JA roles *in vivo *[46]. Among the molecular function categories are glycolysis, energy transfer, cell wall components and water channel activity. Enrichment of cellular components linked to the chloroplast and mitochondrial compartments further strengthen the picture of differences in metabolism between the PGIP over-expressing lines and WT.

Functional groups of differentially expressed genes between the two transgenic lines and WT plants were visualized in MapMan using a mapping file previously developed specifically for the potato TIGR 10 K microarray [41]. As could be expected from the GO enrichment analysis results, the largest changes were seen in different metabolic pathways. A concordance between the lines was seen for groups of genes linked to cell wall modification and degradation, glycolysis, starch synthesis and photosystem-light reactions (Additional file 5).

In order to examine whether the changes in gene expression coupled to an augmented level of *Vvpgip1 *expression seen in this study were similar to the expression pattern observed in other plant systems, the co-expression of *Arabidopsis *genes associated to the orthologous clusters based on sequence similarity were investigated. Strikingly, several gene families affected in the transgenic lines were also found to be co-expressed in *Arabidopsis*, e.g. members of the XTHs, peroxidases UDP epimerases and JAZs (Additional file 1; Additional file 3). The similarities in transcriptional patterns related to *pgip *expression suggest that there is a degree of conservation of these effects between the two species. Furthermore, these similarities between our transgenic system and *Arabidopsis *PGIP expression strengthen the notion that the changes seen in expression pattern is due to *Vvpgip1 *over-expression *per se *and not due to other general properties of PGIPs, such as possible indirect effects resulting from the fact that VvPGIP1 is a secreted, apoplastic protein as discussed as further discussed in Summary and Conclusions.

### Hormone profiling

**Figure 2:**
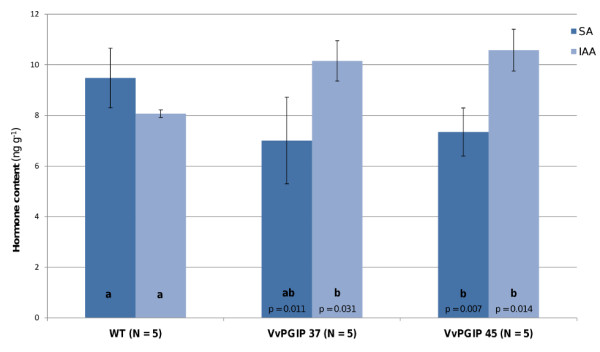
**Phytohormone content**. Salicylic acid (SA) and indole-acetic acid (IAA) content (ng g^-1 ^fresh weight) measured as their corresponding methyl esters in WT and transgenic lines. The total pool of SA (*dark-blue bars*) present in plants as free and methylated forms were analyzed. Both SA and IAA levels (*light-blue bars*) were significantly different from WT in both transgenic lines as indicated by p-values (two-tailed Student's *t*-test). Statistical groups as determined by one-way ANOVA (multi-comparison Bonferroni test, *p <*0.05) are given as letters. No jasmonic acid (total) was detected in the samples. *N *represents the number of biological repeats and *error bars *are given as two times the standard deviation.

**Figure 3:**
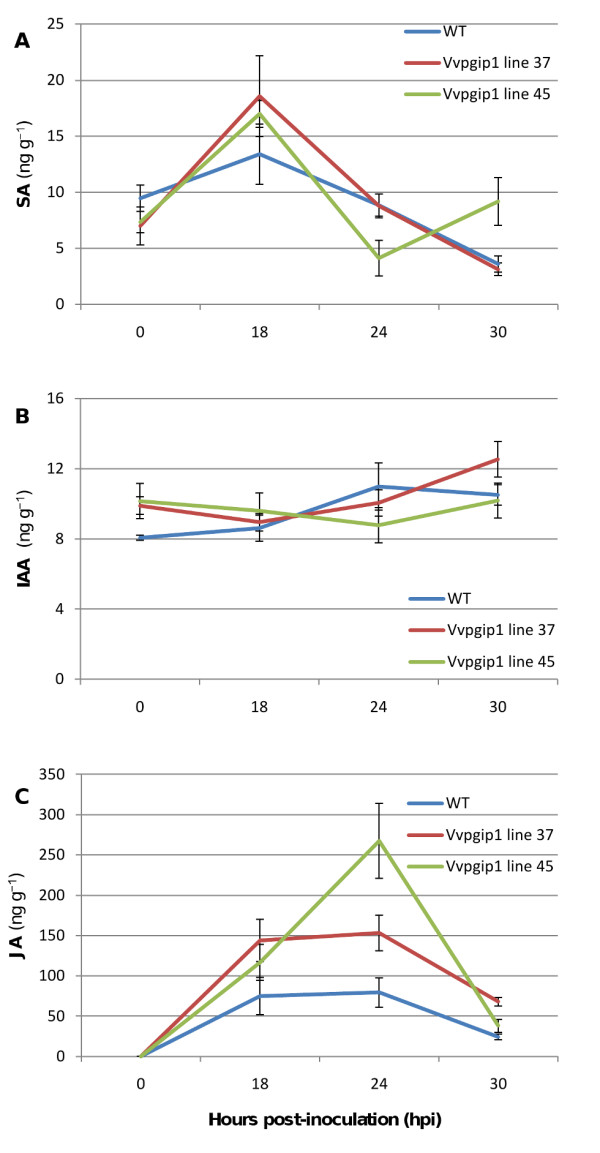
**Phytohormone profiling following *Botrytis *infection**. The hormones (**a**) salicylic acid (SA) (**b**) indole-acetic acid (IAA) and (**c**) jasmonic acid (JA) were measured as their corresponding methyl esters. Two to three plants per time point were used. *Error bars *are given as two times the standard deviation.

As part of the general comparison between the transgenic lines with resistance phenotypes to the susceptible controls, a hormone profile of salicylic acid (SA), indole-acetic acid (IAA) and jasmonic acid (JA) was established (Figure 2). Under the non-infecting conditions used in this study, IAA levels were statistically significantly increased in the transgenic lines, SA levels were slightly lower in the transgenic lines compared to the WT, whereas JA levels were below the detection level in both transgenic lines and WT. Hormone profiling was also performed during a *Botrytis *infection time series (Figure 3). After infection, JA was detectable and both transgenic lines displayed higher JA levels than WT at 18 and 24 h after infection (p = 0.045 at 24 h) at the local lesions. For SA and IAA no definite differences were seen between the groups of transgenic lines and WT following *Botrytis *infection.

Comparing the hormone data with the gene expression analysis, no genes involved in SA biosynthesis were differentially expressed and there was no enrichment of GO terms related to SA regulation. Thus, in spite of SA's involvement in pathogen defense signaling, no strong link between *Vvpgip1 *over-expression and SA and SA-related mechanisms could be established in uninfected or infected tissue. This is in line with an earlier report on PGIP-regulation in *Arabidopsis *[13].

In contrast to SA, indole-acetic acid (IAA) levels without *Botrytis *infection were statistically separable into two groups with the transgenic lines containing higher levels of IAA grouped together (Figure 2). Interestingly, corresponding to the increase in IAA levels there is an over-representation of genes responding to auxin stimulus in the transgenic lines from the microarray data. The down-regulation of the Aux/IAA transcriptional repressors of auxin induced genes, which belong to a large family of transcription factors, could possibly be linked to the over-representation of genes responding to auxin stimulus detected in the *pgip *over-expressing lines. Auxin promotes the degradation of Aux/IAA transcriptional repressors making it possible for auxin response factors (ARFs) to activate the transcription of auxin-responsive genes. Furthermore, increased auxin levels enhance the binding of Aux/IAA proteins to the F-box protein TIR1 leading to ubiquitination and degradation of the Aux/IAA proteins (reviewed in [44]). The expression level of several components of protein degradation and proteolysis were affected, e.g. cullin, a family involved in SCF E3 ubiquitin ligase complexes, ubiquitin proteins, ubiquitin-protein ligase and F-box proteins as well as a number of proteases. The high number of differentially expressed genes involved in protein degradation might be a consequence of changes in hormone signaling, as ubiquitination and targeting for proteosome degradation are common strategies in plant hormone signaling.

Under uninfected conditions JA levels were below the detection level. However, both transgenic lines had a stronger JA response following *Botrytis *infection than WT at the local lesions (Figure 3). On the transcriptional level under un-infecting conditions, *Vvpgip1 *over-expression affected JAZ genes and cyclopentone responsive elements linked to JA regulation and biosynthesis. Like SA, JA was recently suggested to play an important role in signaling leading to Systemic Acquired Response (SAR; [45]). In *Vvpgip *line 37, expression of a LOX gene and a 12-oxophytodienoate-reductase were changed. These enzymes are involved in jasmonate biosynthesis and indicative of an association between PGIPs and JA-mediated signaling. The down-regulation of JAZ genes, which are repressors of JA signaling, indicates that JA signaling pathways might be 'primed' and could explain the quicker response in JA levels observed in the transgenic lines when challenged with a pathogen (Figure 3).

Related to ethylene signaling, s-adenosylmethionine synthetase (SAM), a member of a gene family involved in ethylene biosynthesis, was down-regulated together with an ethylene response factor (AtERF3) belonging to the B1 subfamily of the ERF/AP2 transcription factor family. The latter functions in adaptation to stress and has been shown to be induced by ethylene, JA and pathogens. AtERF1 is also a positive regulator of ET and JA signaling and a possible integration point in the cross-talk between ET and JA signaling pathways (reviewed in [47,48]).

### PGIP expression influences the cell wall by lower XTH activity and increased lignin content

A large number of affected probes matched cell wall-associated genes indicating that cell wall modifications are taking place as an effect of PGIP over-expression. Many differentially expressed probes were linked to lignin and pectin metabolism.

Several tobacco xyloglucan endotransglycosylases (XTHs) representing members of XTH Group I and II were markedly down-regulated. Xyloglucan is the most abundant hemicellulose in dicotyledonous plants and plays a central role in the structure of plant cell walls by cross-linking cellulose microfibrils [49,50] and XTH enzymes are believed to be important for regulation of cell wall strength, extensibility and tissue integrity [51]. XTH Group I and II have *in vitro *been shown to mediate transglucosylation between xyloglucans, in contrast to group III that catalyzes xyloglucan endohydrolysis [52]. It should, however, be noted that the isoforms are grouped according to phylogenetic relationship and that the enzyme activity of all members have not yet been determined.

**Figure 4:**
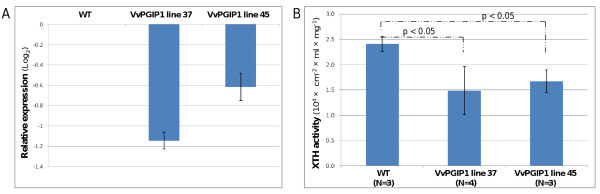
**Expression and activity of XTH**. **a**. Expression of *Nicotiana tabacum *xyloglucan endotransglycosylase (XTH, Genbank Acc AB017025.1) in *Vvpgip1 *lines 37 and 45 relative to the untransformed control (WT). Standard deviation of the mean relative expression of four technical repeats per plant line is shown with *error bars*. **b**. Total XTH activity in tobacco leaves determined by a dot-blot enzyme activity assay. Leaves representing leaf position 3 was used for the assay. A two-tailed Student's *t-test *showed that *p *< 0.05 for both transgenic lines in comparison to WT. N denotes the number of biological repeats.

Because of the important role in cell wall remodeling and marked down-regulation, XTH expression and enzyme activity were investigated further. Indeed, a decrease in XTH expression around the same levels observed in the microarray analysis could be confirmed by RT-qPCR for a tobacco XTH gene belonging to the class I subfamily (Figure 4a). The down-regulation was also confirmed in two additional VvPGIP1 over-expressing lines tested (data not shown). Moreover, a dot-blot enzyme activity assay showed that the general transcriptional down-regulation of XTHs led to a decrease in total XTH activity in leaves of both lines (Figure 4b). Thus, the transcriptional regulation of XTHs had a clear effect on XTH activity, which strengthens the idea that PGIPs have a direct or indirect effect on cell wall modification possibly leading to changes in xyloglucan metabolism.

In tobacco leaves, down-regulation of an *XTH *(*Nt*XET-1) with consequent reduced XTH activity, was previously reported to result in a shift towards xyloglucan with a higher molecular weight, resembling that of older leaves [53]. The authors noted that the cell walls may be strengthened by the reduced turnover and hydrolysis of xyloglucan and it was suggested that the resultant wall strengthening may hold implications for plant-pathogen interactions. XTH activity also increases during fruit ripening and the expression of *V. vinifera *VvXTH1 reaches a maximum at the fully ripe stage when berry softening occurs [54]. Interestingly, *VvXTH1 *expression in grape berries is inversely correlated to *VvPGIP1 *expression, which instead steadily declines until grape berries reach the fully ripe stage [55]. This inversely correlated expression resembles the correlation between *Vvpgip1 *and *XTHs *in the transgenic tobacco. Related to xyloglucan modification, a beta-D-xylosidase with the closest sequence identity to *Arabidopsis *AtXyl4, which is involved in the hydrolysis of the xylan backbone [56], was down-regulated.

Many affected cell wall-associated genes were involved in either lignin or pectin metabolism. The role of lignification in pathogen defense is well documented [57] and cell walls with increased lignin content provide the plant with an effective physical barrier against phytopathogens [58]. Cinnamoyl-CoA reductases (CCR), caffeoyl-CoA O-methyltransferases and beta-glucosidases were differentially expressed and are all involved in monolignol biosynthesis or modification. Monolignol bricks are exported to the cell wall, and then assembled to lignins *in muro *by laccases and peroxidases [59] and changed expression was seen for members of the class III peroxidase family. Recently, it was shown by RNAi silencing that several enzymes in monolignol biosynthesis are crucial for defense against powdery mildew penetration in wheat [60]. In tobacco mutants, down-regulation of a CCR has been shown to lead to changes in the lignin profiles and the syringyl-guaiacyl (S/G) ratio, but not necessarily to altered lignin content [61].

**Figure 5:**
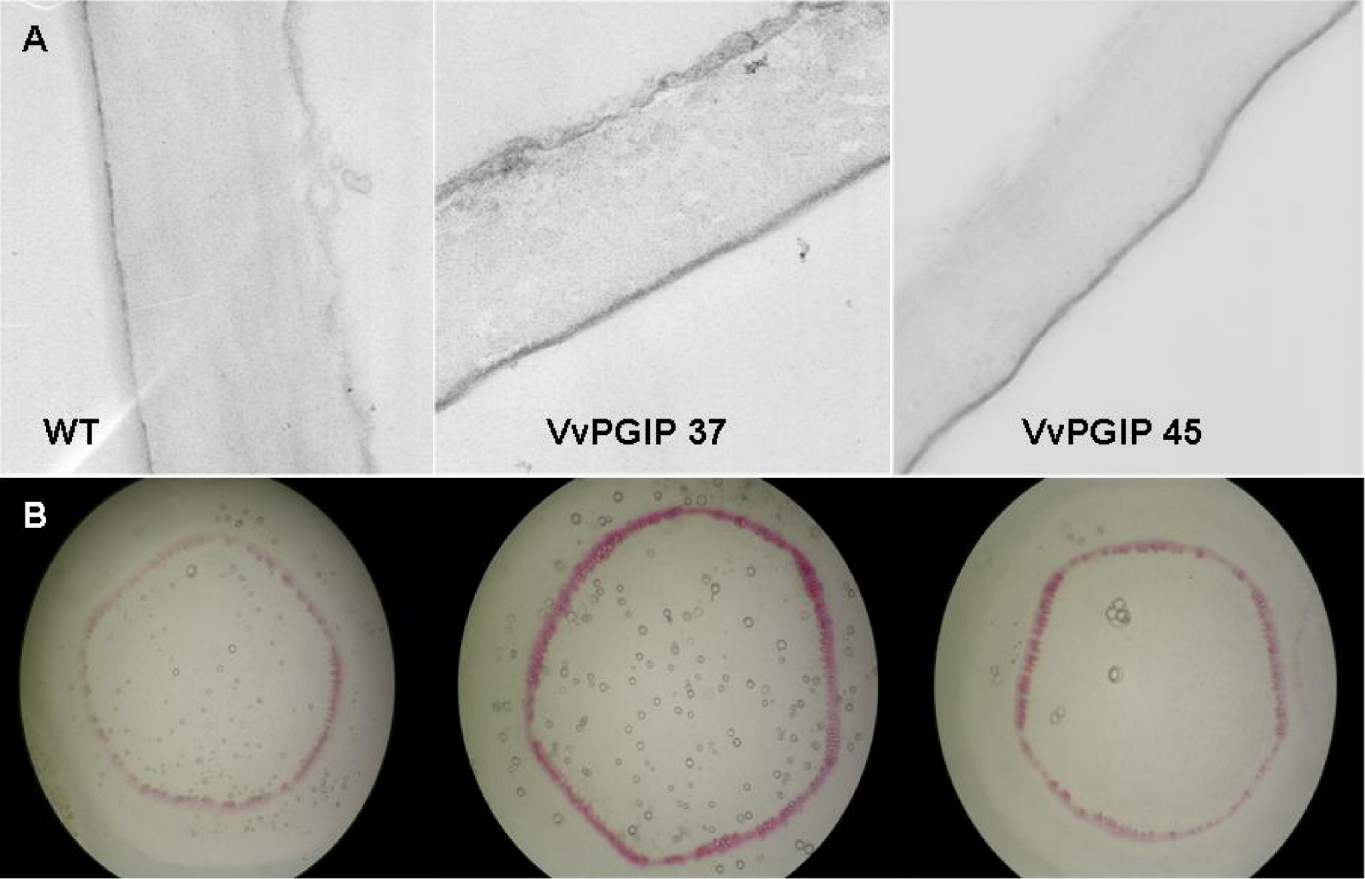
**Lignin content determined by staining**. Leaf and stem sections of WT and transgenic lines (*Vvpgip1 *line 37 and 45) expressing the *Vvpgip1 *transgene. **a**. Sections corresponding smaller parts of the leaf were stained with potassium permanganate and studied by transmission electron microscopy according to [27]. Lignin in the cell walls stain darker in the transgenic lines. **b**. Stem sections were hand cut with a razor and stained with a solution of phloroglucinol according to Ruzin [28]. The intensity of the pink-red stain indicates increased lignin deposition in the secondary xylem. Stem sections were taken from the same internode of plants of the same growth stage; leaves for embedding and sectioning were from the same position on plants of the same age (6-8 leaf stage).

**Figure 6:**
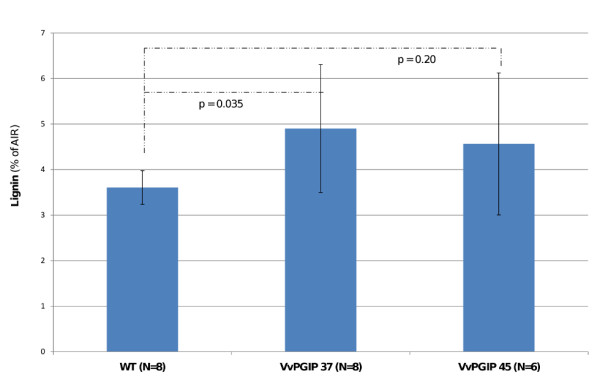
**Cell wall lignin content in leaves**. The lignin content of untransformed control (WT) and transgenic *Vvpgip1 *line 37 and 45 is expressed as the percentage contained in the alcohol-insoluble residue (AIR). Lignin content was determined with the acetyl bromide lignin method, as described in [29]. Statistical analyses (two-tailed Student's *t-test*) could separate the WT from the *Vvpgip1 *line 37 (*p *= 0.035) but not *Vvpgip1 *line 45, for which a similar trend was seen (*p *= 0.20). Standard deviation is given as *error bars*. N represents the number of biological repeats.

Because of the changes seen in gene expression and the known importance of lignin in plant defense, the lignin content was determined. An increased deposition of lignin in leaf and stem tissue of the transgenic lines was observed and by absolute quantification an increased lignin content could be confirmed for *Vvpgip1 *line 37 and a similar trend was seen in line 45 (Figures 5a, b and 6). The increase of lignin in the cell walls of the PGIP-specific resistant lines should impact positively on pathogen resistance.

Even if most evidence for the influence of plant hormones on the plant cell wall is indirect, there is a strong belief that phytohormones influence cell wall biosynthesis and remodeling [62]. Auxin, for example, has an effect on cell wall structure and expansion, acting in combination with brassinosteroids, which was the only other hormone-related GO category enriched in the transgenic lines. Previous work indicates that increased IAA content can be coupled to regulation of *XTH *and the increased deposition of lignin, as was observed in the transgenic lines [63-65]. Catalá et al. [65] reported a down-regulation of a tomato *XTH *gene (*LeXET2*) by auxin and transgenic tobacco lines overproducing IAA exhibited increased lignin content and altered lignin composition [63]. Sitbon et al. [64] suggested that the increased lignin deposition may have resulted from increased peroxidase activity, brought about by increased IAA levels and in the transgenic lines, expression levels of several class III peroxidases changed. However, with the exception of a cytosolic ascorbate peroxidase and in line 45 one peroxidase similar to the *Arabidopsis *peroxidase 53 precursor, the peroxidases are all down-regulated. Whether these changes are a direct result of constitutive over-expression of *pgip *influencing IAA, remains to be seen. Alternatively, the changes seen in cell wall remodeling and metabolism reported here, possibly caused by the interaction of PGIPs in the cell wall, leads to increased IAA-levels.

Among the differentially expressed genes related to pectin biosynthesis and composition was a galacturonosyltransferase-like protein (GATL), which is closely related to other galacturonosyltransferase involved in pectin and/or xylan synthesis. Five *Arabidopsis *GATL mutants were recently characterized and shown to have altered pectin and hemicellulose properties [66]. A putative pectinesterase was also down-regulated. These enzymes modify the degree of methylesterification of pectic homogalacturonan affecting cell wall strengthening (reviewed in [67]). PGIP has been shown to directly interact with pectin *in vitro *by binding a negatively charged homogalacturonan motif [10]. Examining *Arabidopsis *PGIP knock-out and over-expressing lines, a recent report further strengthened the link between PGIP and pectin stability by showing that constitutive PGIP over-expression increased the amount of pectin deposited in the seed coat [17].

Several other down-regulated cell wall-associated genes were involved in sugar metabolism. UDP-glucose epimerases (4-UGE) are known to be involved in the channeling of activated galactose to arabinogalactan proteins [68] and to xyloglucan and pectins [26,69]. Other genes related to cell wall biosynthesis and *in muro *remodeling observed to have altered expression patterns were a beta-galactosidase and a cellulose synthase-like gene.

**Figure 7:**
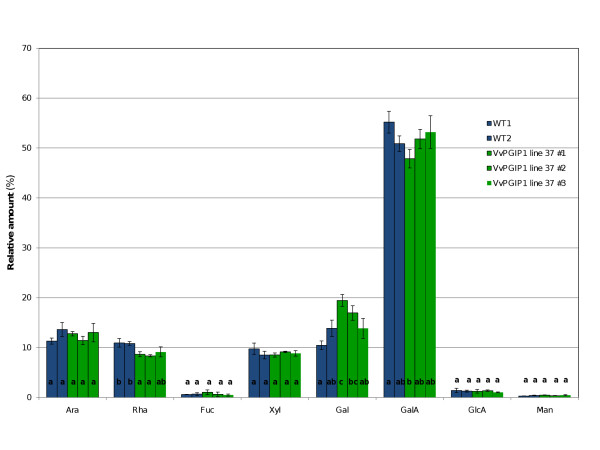
**Cell wall component analysis**. Total cell wall monosaccharide was determined in tobacco leaves (leaf position 3) for two WT and three *Vvpgip1 *line 37 biological repeats. Letters represent statistical groups (within each metabolite) as determined by one-way ANOVA (multi-comparison Bonferroni test, *p *< 0.05).

The overall change in the expression of genes related to cell wall composition and structure observed led to the analysis of the leaf cell wall content of eight monosaccharides in *Vvpgip1 *line 37. However, with the exception of rhamnose, which is associated to rhamnogalacturonan I (RG-I) pectin polysaccharides, the cell wall composition was similar to WT (Figure 7). The differential expression of several genes involved in cell wall remodeling and the confirmation that the composition did not change significantly suggest that cell wall organization, rather than composition is affected by the presence of the PGIP. Future studies will focus on the architecture and cross-linking of cell wall components, specifically with regards to pectin content and composition.

### PGIP expression induces a shift in primary metabolism

Apart from genes related to the cell wall and hormone biosynthesis and signaling, several genes involved in primary metabolism showed a change in expression (Additional file 1). Among these were enzymes involved in glycolysis, the OPP pathway and the TCA cycle. Several genes involved in nitrogen metabolism and catabolism were also affected. Interestingly, some metabolic genes that could be linked to sink-source tissue regulation were identified, e.g. cell wall invertases and an L-asparaginase 4 precursor [70,71]. Cell wall invertases are believed to be key enzymes in the transaction between sink and source tissues and a down-regulation of activity leading to a decrease in hexose sugar availability is an indication of a transformation from sink to source (reviewed in [71]).

Defense mechanisms are energy intensive and during pathogen attack it is necessary for the plant to regulate metabolic pathways in order to deprive the pathogen of energy resources at the same time as it recruits energy for its own defense response (reviewed in [72]). Our results indicate that the *Vvpgip1 *over-expressing lines have adjustments of the primary metabolism even before pathogen attack which might be indicative of a 'primed-like' state. Alternatively, the changes seen in primary metabolism might also be linked to the changes observed in cell wall remodeling and metabolism, which probably affects energy demand and biosynthesis of various building blocks needed. These aspects need further investigation, specifically also in the presence of an infecting pathogen.

## Summary and conclusions

Taken together, there is clear evidence that transgenic lines over-expressing *Vvpgip1 *have altered cell wall properties compared to their untransformed counterparts, even in the absence of pathogen infection. In addition, increased levels of auxin in uninfected tissue and an amplified JA response following *Botrytis *infection were seen. Interestingly, there were subtle changes in the transcription of a large number of genes representing different distinct functional sets, notably related to cell wall functions, primary metabolism and stress responses. Among the more markedly down-regulated genes were *XTH*s, and we could show that the decrease in *XTH *expression led to a lowered XTH activity. The alteration of XTHs, together with an increased deposition of lignin also observed, are possible contributors to the reduced *Botrytis *susceptibility observed in these PGIP-specific resistant lines.

The current hypothesis is that PGIP's involvement in plant defense is limited to the inhibition of ePGs limiting tissue maceration and necrosis. Also, following inhibition of ePG by PGIP, it is believed that the lifetime of molecules with elicitor activity towards the activation of plant defenses are extended [7]. But, since the conditions primarily studied here were with the pathogen absent, neither the inhibition of ePG nor the extension of the lifetime of oligogalacturonides were involved. PGIP may directly influence defense responses in the plant possibly by strengthening the cell walls; whether by virtue of its structural features, which contains a LRR structure shared with many receptor involved in pathogen recognition or its integration in the cell wall.

However, at this stage we cannot exclude that the effects of the *Vvpgip1 *over-expression observed are due to properties specific to VvPGIP1. Putatively, these could originate from more general properties of the protein, e.g., as an effect of its cellular transportation and presence in the apoplast. Still, the cross-species co-expression analysis shows that important genes like members of XTHs, peroxidases, UDP epimerases and JAZs are selectively co-expressed also with PGIPs in *Arabidopsis*, and thus gives some evidence that these groups of genes are specifically affected by the PGIP expression levels.

Lately, other evidence indicating a broader role of PGIPs has been presented. For example, studies have shown that PGIP-encoding gene regulates floral organ number in rice, that the expression of a pea PGIP affects the resistance to nematode invasion without the detection of ePGs in either organism and that PGIP expression influences seed imbibition [16,17,72]. In addition to the inhibition of ePGs and subsequent signaling events, these observations of PGIP's effects on plant development and pathogen resistance together with the work presented in this manuscript, are shedding new light on the *in planta *roles of PGIP.

## Competing interests

The authors declare that they have no competing interests.

## Authors' contributions

EA did the microarray data and statistical analyses. JVWB performed the microarrays, qPCR and hormone and lignin profiling experiments. DJ constructed the bioinformatical workflow and graph structures. ENO determined the cell wall composition and CS did the XTH enzyme activity assay. KJD participated in the design of the microarray study. MAV conceived the study.

EA, JVWB, DJ and MAV drafted the manuscript. All authors read and approved of the final manuscript.

## Supplementary Material

Additional file 1 **Table of probes displaying differential expression**. Table of all probes displaying differential expression. Differential expression (Log2) between control plants and *Vvpgip1*-transformed lines 37 and 45 (FDR **<**0.05). Probes are divided into orthologous clusters based on sequence similarity (see Materials and Methods). Functional category, name, an arbitrary set identification number and number of hybridizing transcripts are given for each orthologous cluster. The sequence similarity (%) and amino acid length of similar sequence for the top hit in *Arabidopsis* of each probe sequence is also included. If no match was found in *Arabidopsis* the top hit for rice was instead given. The descriptions presented are derived from the top hits. The level of co-expression of *Arabidopsis* genes associated to two or more of the orthologous clusters based on sequence similarity are given as Pearson coefficients correlations with different stringency (0.05, 0.1 or 0.2). Asterisk denotes that only a trend (p < 0.15) was seen in *Vvpgip1* line 45. Click here for file

Additional file 2 **Updated Gene Ontology (GO terms) associated with each probe on the TIGR 10 k microarray**. Updated GO terms associated with each probe on the TIGR 10 k microarray. GO terms were updated with information retrieved from nine plant species with the help of a graph structure. The table is compatible for use with GOEast if saved as a tab-delimited file. Click here for file

Additional file 3 **A selection of differentially expressed genes**. Probes are divided into orthologous clusters based on sequence similarity and expression ratios are given in Log2-scale. Top hits based on sequence similarity are given for either *Arabidopsis* or rice and includes the corresponding gene description. Related citations are given in the heading literature. Shaded orthologous clusters indicate that associated *Arabidopsis* genes based on sequence identity were co-expressed (Pearson coefficient correlation < 0.2) with one or more additional *Arabidopsis* genes linked to the orthologous clusters. Asterisks denote that only a similar trend for differential expression was observed in *Vvpgip* 1 line 45 (FDR < 0.15). For more details on constructions of orthologous clusters and cross-species co-expression analysis, see Materials and Methods. Click here for file

Additional file 4 **Gene ontology enrichments**. GO enrichment of probes showing significant difference in signal intensity in *Vvpgip1* line 37 and 45 in comparison to WT. (A) Biological process (B) Cellular compartment and (C) Molecular function. The gene ontology maps were generated in GOEast [39]. Enriched terms are colored in yellow and the intensity of the color yellow denotes the level of enrichment. Red arrows stand for relationship between two enriched GO terms, black solid arrows stand for relationship between enriched and not enriched terms and black dashed arrows stand for relationship between two not enriched GO terms. Click here for file

Additional file 5 **MapMan overview of metabolic categories comparing *Vvpgip1* line 37 and 45**. A MapMan mapping file adopted for the TIGR 10 K potato microarray was used [41]. Expression values were filtered after FDR-adjustment (p < 0.05). Click here for file
