# Supplementary material for: Constitutive expression of a grapevine polygalacturonase-inhibiting protein affects gene expression and cell wall properties in uninfected tobacco
Source: BMC Res Notes. 2011 Nov 13;4:493. doi: 10.1186/1756-0500-4-493 (PMC3339426; doi:10.1186/1756-0500-4-493)
Supplement: Additional file 3 — A selection of differentially expressed genes. Probes are divided into orthologous clusters based on sequence similarity and expression ratios are given in Log2-scale. Top hits based on sequence similarity are given for either Arabidopsis or rice and includes the corresponding gene description. Related citations are given in the heading literature. Shaded orthologous clusters indicate that associated Arabidopsis genes based on sequence identity were co-expressed (Pearson coefficient correlation < 0.2) with one or more additional Arabidopsis genes linked to the orthologous clusters. Asterisks denote that only a similar trend for differential expression was observed in Vvpgip 1 line 45 (FDR < 0.15). For more details on constructions of orthologous clusters and cross-species co-expression analysis, see Materials and Methods. [file 1756-0500-4-493-S3.DOC]

| **Probe cluster number and name** | | | **Probe IDs** | **Line 37** | **Line 45** | **AtID or OsID** | **Description** |
| --- | --- | --- | --- | --- | --- | --- | --- |
| ***Hormone biosynthesis, transport and regulation*** | | | | | | | |
|  | | ***4. S-adenosylmethionine synthetase*** | STMGR58 | -0.201 | -2.455 | AT1G02500 | S-adenosylmethionine synthetase (SAM1) |
|  | | ***67. JAZs*** | STMCV86 | -0.318 | -0.529 | AT3G17860 | jasmonate-zim-domain proteins (JAZ) |
|  | | ***136. auxin inducible transcription factors*** | STMGB57 | -0.276 | -0.554 | AT3G04730 | early auxin-induced (IAA16) |
|  | |  | STMDP46 | -0.284 | -0.386 | AT1G04240 | SHY2/IAA3 |
|  | | ***160. JAZs*** | STMCZ36 | -0.284 | -0.419 | AT3G17860 | jasmonate-zim-domain proteins (JAZ) |
|  | | ***172. amino acid transporters*** | STMGC39 | -0.453 | -0.504 | AT3G56200 | amino acid transporter family protein |
|  | | ***261. ethylene response factor TF*** | STMEZ35 | -0.232 | -0.293 | AT1G50640 | ERF/AP2 transcription factor |
|  | | ***273. putative gibberellin receptor*** | STMGA83 | -0.17 | -0.407 | AT3G63010 | gibberellin (GA) receptor |
|  | |  |  |  |  |  |  |
| ***Primary carbon metabolism and nitrogen metabolism and regulation*** | | | | | |  |  |
|  | ***18. fructose-bisphosphate aldolases*** | | STMED26 | 0.357 | 0.401 | AT2G36460 | fructose-bisphosphate aldolase |
|  | ***56. L-asparaginase 1 precursor*** | | STMGG26 | -0.195 | -1.121 | AT5G08100 | putative L-asparaginase 4 precursor |
|  | ***76. 3-methylcrotonyl-CoA carboxylase 1*** | | STMIT78 | -0.346 | -0.791 | AT1G03090 | 3-methylcrotonyl-CoA carboxylase 1 |
|  | ***98. PSII-S (CP22)*** | | STMCD24 | 0.333 | 0.324 | AT1G44575 | Encoding PSII-S (CP22) |
|  | ***105. pyruvate kinases*** | | STMDG22 | -0.694 | -0.787 | AT3G22960 | chloroplast pyruvate kinase alpha subunit |
|  | ***117. glutamate dehydrogenases*** | | STMJL47 | -0.538 | -0.533 | AT1G51720 | putative GDH2 (GLUTAMATE DEHYDROGENASE 2) |
|  |  | | STMIX11 | -0.324 | -0.594 | AT5G18170 | putative GDH1 (GLUTAMATE DEHYDROGENASE 1) |
|  | ***118. possible acetyl-CoA carboxylase*** | | STMIX24 | -0.303 | -0.5 | AT2G38040 | acetyl-CoA carboxylase |
|  | ***122. chlorophyll binding protein D1*** | | STMCV20 | -0.591 | -2.063 | ATCG00020 | PSII 32 KDa protein (psbA) |
|  | ***148. glyceraldehyde-3-phosphate dehydrogenase*** | | STMDC74 | 0.275 | 0.362 | AT1G13440 | cyt. glyceraldehyde-3-phosphate dehydrogenase (GAPC) |
|  |  | | STMDH57 | 0.359 | 0.315 | AT1G13440 | cyt. glyceraldehyde-3-phosphate dehydrogenase (GAPC) |
|  | ***169. mitochondrial ADP/ATP carrier*** | | STMDR46 | -0.902 | -0.895 | AT3G08580 | mitochondrial ADP/ATP carrier |
|  | ***171. acyl-CoA binding protein*** | | STMGT25 | -0.198 | -1.346 | AT3G05420 | acyl-CoA binding |
|  | ***245. putative diaminopimelate decarboxylase 2*** | | STMDV29 | -0.164 | -0.469 | AT5G11880 | putative diaminopimelate decarboxylase |
|  | ***258. putative ATPase*** | | STMGG30 | -0.252 | -1.334 | AT3G47760 | ATPase |
|  |  | |  |  |  |  |  |
| ***Cell wall metabolism and modification*** | | |  |  |  |  |  |
|  | ***1. UDP-glucose epimerases*** | | STMIA83 | -0.282 | -0.364 | AT1G12780 | UDP-glucose (UGE1) |
|  | ***3. beta-d-xylosidase*** | | STMEA15 | -0.213 | -0.447 | AT5G64570 | beta-xylosidase 4 (XYL4) |
|  | ***6. Cell wall invertases*** | | STMCF35* | -0.233 | -0.289 | AT3G52600 | similar to cell wall invertase 4 |
|  | ***24. galacturonosyltransferase-like proteins*** | | STMCE40 | -0.195 | -0.388 | AT1G13250 | galacturonosyltransferase-like 3 (GATL3) |
|  | ***32. putative trehalose biosynthesis enzymes*** | | STMIU93 | -0.25 | -0.435 | AT1G06410 | trehalose-phosphatase/synthase 7 |
|  | ***41. putative pectinesterase family proteins*** | | STMJE46* | -0.469 | -0.443 | AT5G09760 | pectinesterase family protein |
|  | **63a. xyloglucan endotransglucosylase/hydrolase** | | STMDB57 | -1.007 | -0.87 | AT5G57550 | xyloglucan endotransglycosylase-related protein, XTH25 |
|  |  | | STMJB14 | -0.731 | -0.9 | AT5G57560 | xyloglucan endotransglucosylase/hydrolase protein,XTH22 |
|  |  | | STMEI79* | -0.507 | -0.369 | AT5G13870 | xyloglucan endotransglucosylase/hydrolase protein, XTH5 |
|  |  | | STMJJ17 | -0.906 | -0.992 | AT4G25810 | xyloglucan endotransglycosylase-related protein, XTH23 |
|  |  | | STMEP06 | -1.162 | -1.321 | AT3G23730 | xyloglucan endotransglucosylase/hydrolase protein, XTH16 |
|  |  | | STMEQ95 | -1.018 | -1.106 | AT4G14130 | xyloglucan endotransglycosylase-related protein, XTH7 |
|  | ***63b. cytosolic ascorbate peroxidase*** | | STMGH65 | -0.587 | -0.544 | AT1G07890 | cytosolic ascorbate peroxidase (APX1) |
|  |  | | STMHU05 | 0.281 | 0.319 | AT3G09640 | cytosolic ascorbate peroxidase (APX2) |
|  | ***63c. NRPs*** | | STMGI55* | 0.242 | 0.205 | AT1G18800 | nap1-related protein 2 (NRP2) |
|  | ***121. putative O-methyltransferase family 2 prot*** | | STMGA58 | -0.482 | -1.087 | AT4G35150 | similar to O-methyltransferase family 2 protein |
|  | ***142. zinc finger family proteins*** | | STMGB38 | -0.181 | -0.322 | AT5G20910 | zinc finger (C3HC4-type RING finger) family protein |
|  | ***143. putative cinnamoyl-CoA reductase*** | | STMDZ37 | -0.186 | -1.504 | AT5G58490 | similar to cinnamoyl-CoA reductase family |
|  | ***149. fasciclin-like arabinogalactan-proteins*** | | STMIJ79 | -0.464 | -1.136 | AT1G03870 | fasciclin-like arabinogalactan-protein 9 (Fla9) |
|  | ***154a. DEAD-box ATP-dependent RNA helicase*** | | STMJO48 | 0.27 | 0.475 | AT3G09720 | putative DEAD/DEAH box helicase |
|  | ***154b. UDP-D-glucuronate 4-epimerase*** | | STMDP31 | -0.321 | -0.474 | AT4G30440 | UDP-D-glucuronate 4-epimerase (GAE1) |
|  | ***244. putative beta-galactosidase*** | | STMGR50 | -0.449 | -0.89 | AT4G36360 | putative beta-galactosidase (BGAL3) |
|  | ***260. cellulose synthase-like G3*** | | STMJM75* | 0.243 | 0.311 | AT4G23990 | Cellulose synthase-like G3 |
|  | ***277. putative peroxidase precursor*** | | STMGB54 | -0.23 | -0.556 | AT2G18980 | Identical to Peroxidase 16 precursor (PER16) |
|  |  | |  |  |  |  |  |
| ***Signaling, defense and stress response*** | | |  |  |  |  |  |
|  | ***34. calreticulin*** | | STMJA91* | 0.526 | 0.275 | AT1G56340 | calreticulin 1 (Crt1) |
|  | ***78. putative protein kinase family*** | | STMHE11 | -0.192 | -0.286 | AT3G13670 | protein kinase family protein |
|  | ***168. putative DnaJ chaperon*** | | STMCH83 | -0.821 | -0.258 | AT3G44110 | co-chaperon DNAJ protein |
|  | ***176. patatin-like proteins*** | | STMGD80 | -0.394 | -1.223 | AT2G39220 | similar to Patatin-like protein 7 |
|  | ***204. putative protein kinase*** | | STMGS70 | 0.264 | 0.374 | AT3G17850 | putative protein kinase |
|  | ***210. calcium-dependent protein kinase*** | | STMGB58 | -0.165 | -0.579 | AT2G46700 | putative calcium-dependent protein kinase (CDPK) |
|  | ***211. putative extracellular dermal glycoprotein*** | | STMJF08 | -0.516 | -0.799 | AT1G03230 | putative extracellular dermal glycoprotein |
|  | ***212. HSP90-like protein*** | | STMEG24 | 0.531 | 0.413 | AT4G24190 | HSP90-like protein |
|  | ***219. dnaJ proteins*** | | STMGI18 | -0.359 | -0.59 | AT4G02100 | similar to DNAJ heat shock |
|  | ***224. putative calnexin*** | | STMEN67 | 0.4 | 0.292 | AT5G61790 | calnexin 1 |
|  | ***228. TIR-NBS-LRR class proteins*** | | STMHR33* | -0.401 | -0.359 | AT5G17680 | similar to disease resistance protein (TIR-NBS-LRR class) |
|  | ***270. NPR1-like*** | | STMIM01 | -0.302 | -0.561 | AT5G63160 | btb and taz domain protein 2 (BT2) |
|  | ***275. plasma membrane polypeptide*** | | STMEZ77 | -0.669 | -0.707 | AT4G20260 | similar to DREPP2 protein |
|  | ***276. remorin*** | | STMHV95 | -0.8 | -0.589 | AT3G48940 | remorin family protein |
|  |  | |  |  |  |  |  |
| ***Transport*** | | |  |  |  |  |  |
|  | ***22. equilibrative nucleoside transporters*** | | STMGS84 | -0.66 | -1.733 | AT4G05120 | equilibrative nucleoside transporter (ENT3) |
|  | ***64. sec24-like proteins*** | | STMGF51 | -0.32 | -2.581 | AT3G44340 | homologous to yeast and animal Sec24 proteins |
|  | ***101. aquaporins (plasma membrane intrinsic proteins)*** | | STMCS35 | -0.518 | -0.563 | AT4G23400 | aquaporin PIP1;5 |
|  |  | | STMGW25 | -0.421 | -0.625 | AT1G01620 | aquaporin PIP1;3 |
|  |  | | STMIQ12 | -0.41 | -0.467 | AT2G37180 | aquaporin PIP2;3 |
|  |  | | STMCJ96 | -0.49 | -0.813 | AT3G53420 | aquaporin PIP2;1 |
|  |  | | STMEQ06 | -0.545 | -0.51 | AT2G37170 | aquaporin PIP2;2 |
|  |  | | STMEK85 | -0.317 | -0.598 | AT4G35100 | aquaporin PIP2;8 |
|  |  | | STMCQ72 | -0.345 | -0.424 | AT4G00430 | aquaporin PIP1;4 |
|  | ***181. putative cation/H+ antiporter*** | | STMHE26 | -0.618 | -0.413 | AT3G51860 | calcium exchanger family (CAX3) |
|  | ***251. putative vesicle-associated membrane prot*** | | STMDS85* | 0.319 | 0.282 | AT5G47180 | VAMP family protein |
|  | ***266. aquaporins (tonoplast intrinsic proteins)*** | | STMIV08 | -0.402 | -0.704 | AT3G16240 | aquaporin TIP2;1 |
|  |  | | STMIH11 | -0.368 | -0.445 | AT5G47450 | aquaporin TIP2;3 |
|  |  | |  |  |  |  |  |
|  | ***Additional groups of differentially expressed genes*** | |  |  |  |  |  |
|  | ***2. ubiquitin related proteins*** | | STMJK08 | 0.293 | 0.348 | AT2G36170 | ubiquitin (UBQ16) |
|  |  | | STMEV28 | 0.294 | 0.31 | AT3G52590 | ubiquitin extension protein (UBQ1) |
|  | ***21. ubiquitin-protein ligases*** | | STMJJ04 | -0.538 | -0.275 | AT2G02760 | ubiquitin conjugating enzyme (UBC2) |
|  | ***70. digalactosyl diacylglycerol deficient protein*** | | STMEZ94 | -0.338 | -0.749 | AT3G11670 | digalactosyl diacylglycerol deficient 1 (DGD1) |
|  | ***207. put. transcriptionally controlled tumor protein*** | | STMDH92 | -0.506 | -0.534 | AT3G16640 | similar to transcriptionally controlled tumor protein (TCTP) |
|  |  | | STMGQ05 | -0.787 | -0.545 | AT3G16640 | similar to transcriptionally controlled tumor protein (TCTP) |
|  | ***250. cullin*** | | STMGC36 | -0.199 | -0.624 | AT5G46210 | cullin4 (CUL4) |
