# Supplementary figures and images for: Constitutive expression of a grapevine polygalacturonase-inhibiting protein affects gene expression and cell wall properties in uninfected tobacco
Source: BMC Res Notes. 2011 Nov 13;4:493. doi: 10.1186/1756-0500-4-493 (PMC3339426; doi:10.1186/1756-0500-4-493)

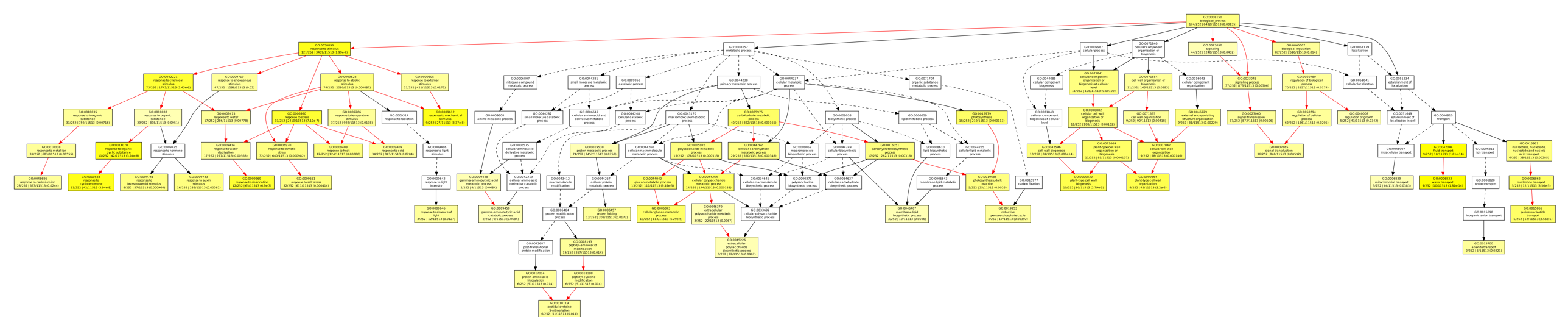

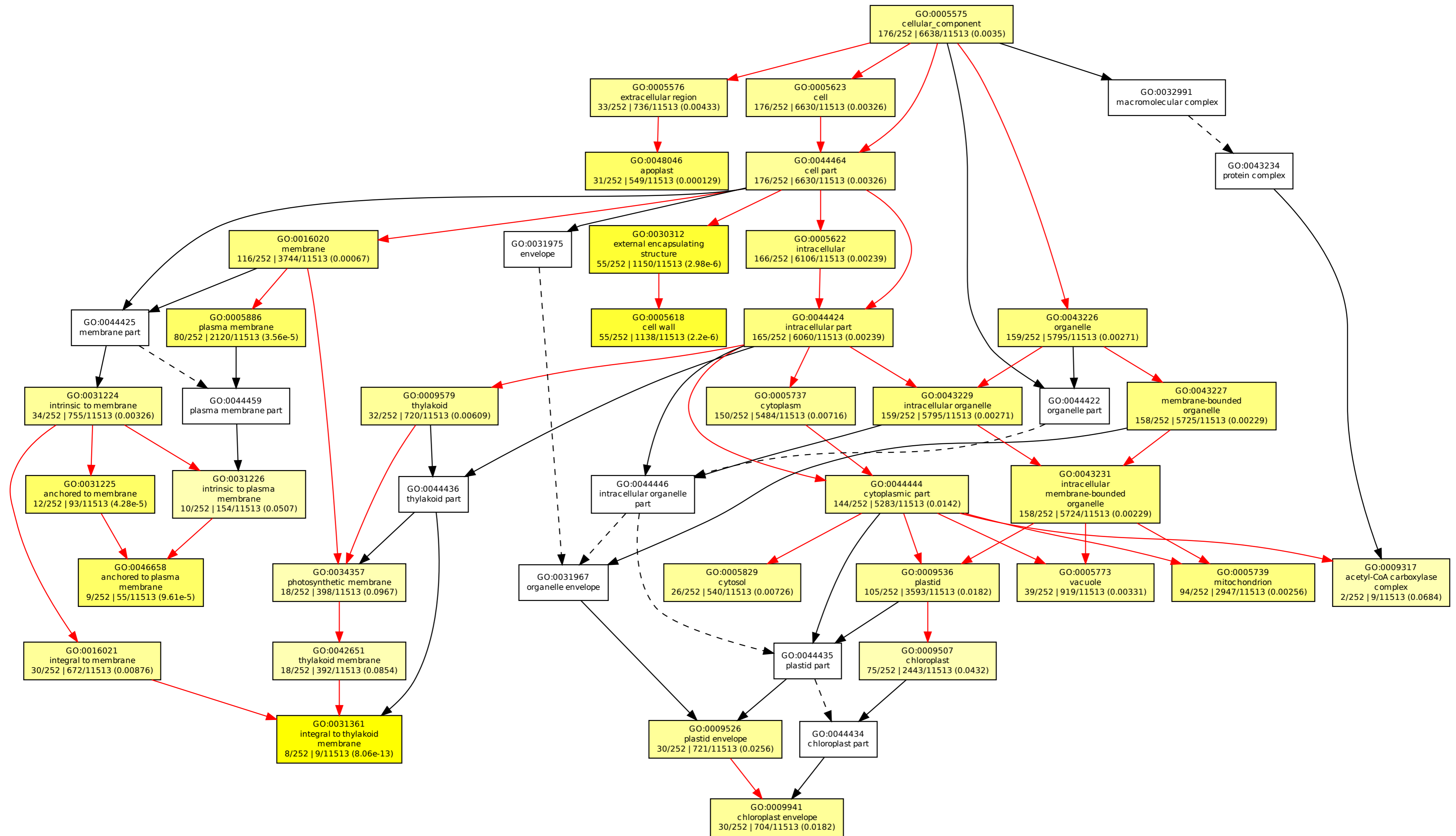

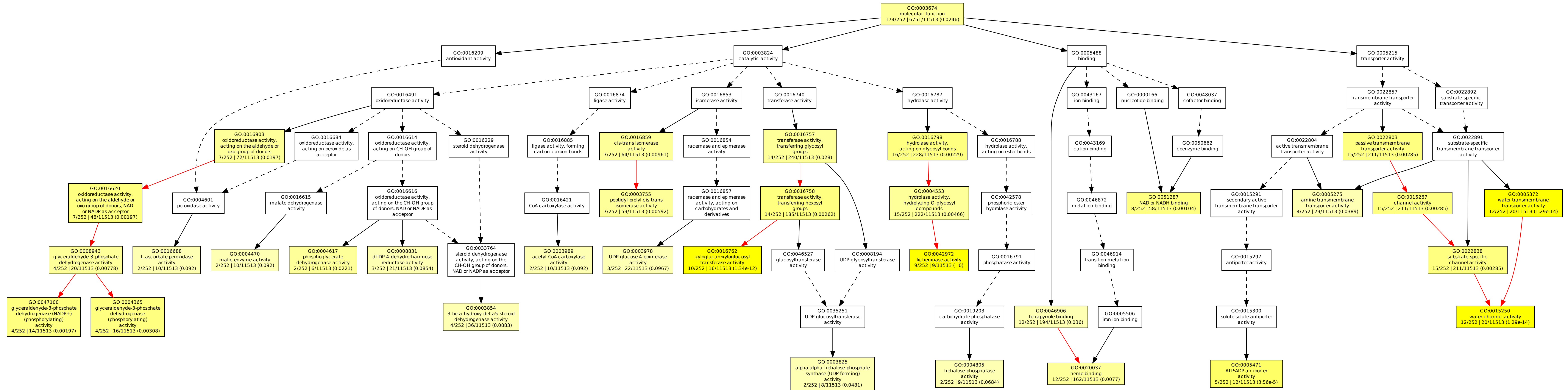

Supplement: Additional file 4 — Gene ontology enrichments. GO enrichment of probes showing significant difference in signal intensity in Vvpgip1 line 37 and 45 in comparison to WT. (A) Biological process (B) Cellular compartment and (C) Molecular function. The gene ontology maps were generated in GOEast [39]. Enriched terms are colored in yellow and the intensity of the color yellow denotes the level of enrichment. Red arrows stand for relationship between two enriched GO terms, black solid arrows stand for relationship between enriched and not enriched terms and black dashed arrows stand for relationship between two not enriched GO terms. [file 1756-0500-4-493-S4.PDF]
