# Supplementary material for: Constitutive expression of a grapevine polygalacturonase-inhibiting protein affects gene expression and cell wall properties in uninfected tobacco
Source: BMC Res Notes. 2011 Nov 13;4:493. doi: 10.1186/1756-0500-4-493 (PMC3339426; doi:10.1186/1756-0500-4-493)
Supplement: Additional file 5 — MapMan overview of metabolic categories comparing Vvpgip1 line 37 and 45. A MapMan mapping file adopted for the TIGR 10 K potato microarray was used [41]. Expression values were filtered after FDR-adjustment (p < 0.05). [file 1756-0500-4-493-S5.PDF]

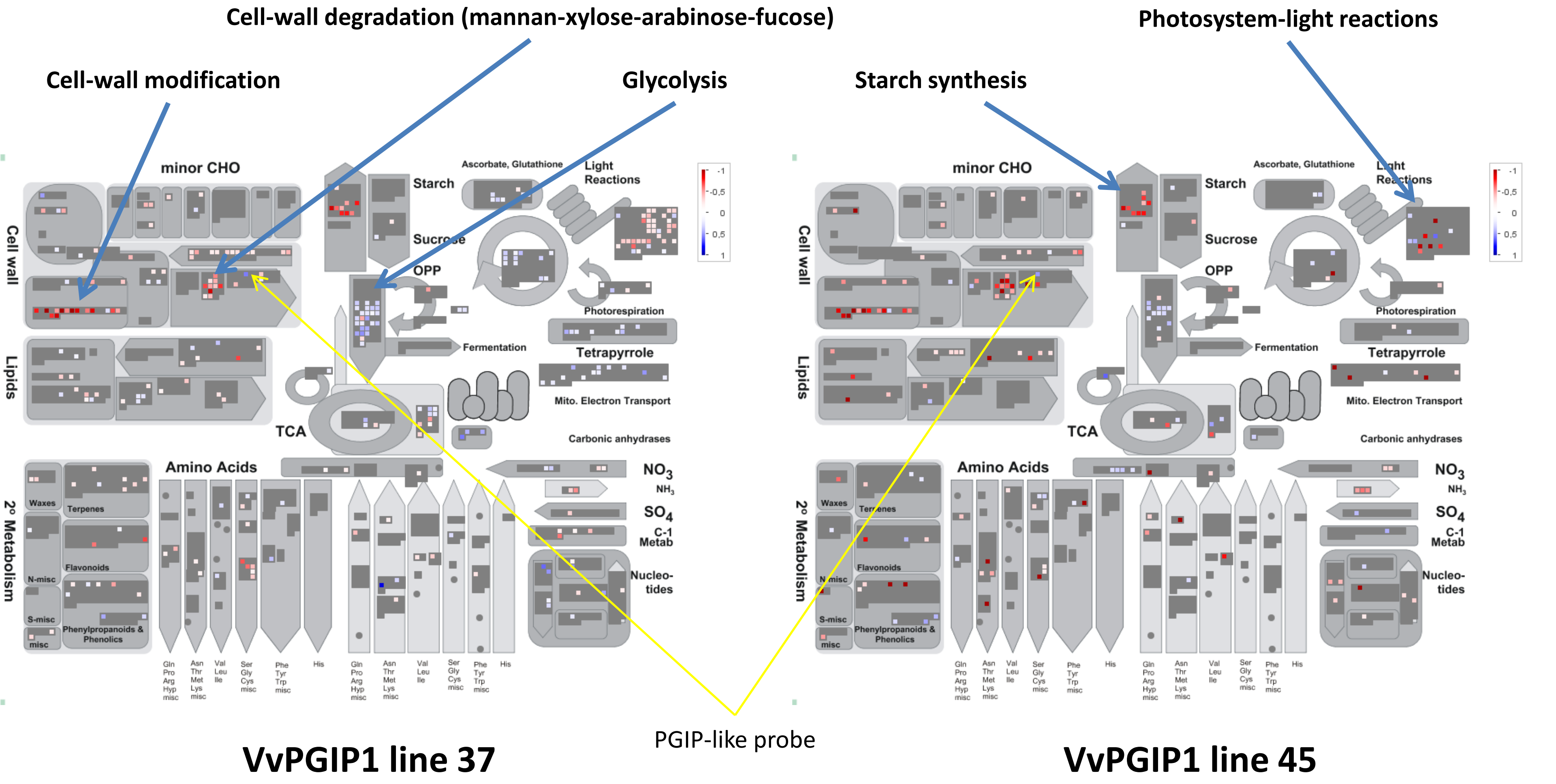

Cell-wall degradation (mannan-xylose-arabinose-fucose)
Photosystem-light reactions
Cell-wall modification
Glycolysis
Starch synthesis
minor CHO
Cell wall
Lipids
Starch
Sucrose
OPP
Ascorbate, Glutathione
Light Reactions
Photorespiration
Fermentation
Tetrapyrrole
Mito. Electron Transport
Carbonic anhydrases
TCA
Amino Acids
2° Metabolism
Waxes
Terpenes
Flavonoids
N-misc
S-misc
misc
Phenylpropanoids & Phenolics
NO3
NH3
SO4
C-1 Metab
Nucleo-tides
Gln Pro Arg Hyp misc
Asn Thr Met Lys misc
Val Leu Ile
Ser Gly Cys misc
Phe Tyr Trp misc
His
-1
-0,5
0
0,5
1
PGIP-like probe
VvPGIP1 line 37
VvPGIP1 line 45
